# QCBCT-NET for direct measurement of bone mineral density from quantitative cone-beam CT: a human skull phantom study

**DOI:** 10.1038/s41598-021-94359-2

**Published:** 2021-07-23

**Authors:** Tae-Hoon Yong, Su Yang, Sang-Jeong Lee, Chansoo Park, Jo-Eun Kim, Kyung-Hoe Huh, Sam-Sun Lee, Min-Suk Heo, Won-Jin Yi

**Affiliations:** 1 Department of Applied Bioengineering, Graduate School of Convergence Science and Technology, Seoul National University, Seoul, Korea; 2 Dental Research Institute, Seoul National University, Seoul, Korea; 3 Department of Oral and Maxillofacial Radiology, School of Dentistry, Seoul National University, Seoul, Korea; 4 Department of Oral and Maxillofacial Radiology, Seoul National University Dental Hospital, Seoul, Korea; 5 Department of Oral and Maxillofacial Radiology and Dental Research Institute, School of Dentistry, Seoul National University, Seoul, Korea

**Keywords:** Osteoporosis, Dental diseases, Medical imaging

## Abstract

The purpose of this study was to directly and quantitatively measure BMD from Cone-beam CT (CBCT) images by enhancing the linearity and uniformity of the bone intensities based on a hybrid deep-learning model (QCBCT-NET) of combining the generative adversarial network (Cycle-GAN) and U-Net, and to compare the bone images enhanced by the QCBCT-NET with those by Cycle-GAN and U-Net. We used two phantoms of human skulls encased in acrylic, one for the training and validation datasets, and the other for the test dataset. We proposed the QCBCT-NET consisting of Cycle-GAN with residual blocks and a multi-channel U-Net using paired training data of quantitative CT (QCT) and CBCT images. The BMD images produced by QCBCT-NET significantly outperformed the images produced by the Cycle-GAN or the U-Net in mean absolute difference (MAD), peak signal to noise ratio (PSNR), normalized cross-correlation (NCC), structural similarity (SSIM), and linearity when compared to the original QCT image. The QCBCT-NET improved the contrast of the bone images by reflecting the original BMD distribution of the QCT image locally using the Cycle-GAN, and also spatial uniformity of the bone images by globally suppressing image artifacts and noise using the two-channel U-Net. The QCBCT-NET substantially enhanced the linearity, uniformity, and contrast as well as the anatomical and quantitative accuracy of the bone images, and demonstrated more accuracy than the Cycle-GAN and the U-Net for quantitatively measuring BMD in CBCT.

## Introduction

Trabecular bone density, a determinant of bone strength, is important for the diagnosis of bone quality in bone diseases^1,2^. Bone mineral density (BMD) measurements are a direct method of estimating human bone mass for diagnosing osteoporosis and predicting future fracture risk^3,4^. Generally, volumetric BMD can be assessed quantitatively through the calibration of Hounsfield Units (HU) in CT, which is a method known as quantitative CT (QCT)^5,6^. The multi-detector CT (MDCT) with rapid acquisition of 3D volume images enables QCT to be applied to clinically important sites for assessing BMD^7^.

For dental implant treatment, precise in vivo measurement of alveolar bone quality is very important in determining the primary stability of dental implants^8^. Therefore, the alveolar bone quality of the implant site needs to be measured before surgery to determine whether the BMD is sufficient to support the implant^9^. Recently, cone-beam CT (CBCT) systems have been widely used for dental treatment and planning as they offer many advantages over MDCTs, including a lower radiation dose to the patient, shorter acquisition times, better resolution, and greater detail^10–15^. However, the voxel intensity values in CBCT systems are arbitrary, and do not allow for the assessment of bone quality as the systems do not correctly show HUs^16–20^. The ability of the CBCT to assess the bone density is limited as the HUs derived from CBCT data is clearly different from that of MDCT data^5,17–19,21^. Several studies have been performed to resolve the discrepancy in HUs between MDCT and CBCT data^15–17,22^. Some studies investigated the relationship between CBCT voxel intensity values and MDCT HUs using a BMD calibration phantom with material inserts of different attenuation coefficients^17,23–27^. These studies showed that the use of the phantoms in CBCT scanners would be difficult for correlating CBCT voxel intensities with HUs because of the non-uniformity of the measurements and the nonlinear relationship between CBCT voxel intensities and HUs^15^.

CBCTs have also been widely used for accurate patient setups in image-guided radiation therapy^28^. Many methods for correcting CBCT images with high quality have been proposed to produce quantitative CBCTs in the radiation therapy field, which do not require a calibration phantom during an object scan. These methods can be classified as hardware corrections such as anti-scatter grids, and model-based methods using Monte Carlo techniques to model the scatter to CBCT projections^29–34^. Recently, the generative adversarial network (GAN), a deep neural network model, has shown state-of-the-art performance in many image processing tasks^28,35,36^. The GAN is composed of two networks trained simultaneously with one focused on image generation and the other on discrimination. The GAN has the capability of data generation without explicitly modelling the probability density function^37^. In one study, a deep learning-based method using a modified GAN improved image quality for generating corrected CBCT images, which integrated a residual block concept into a Cycle-GAN framework^38^. Moreover, the U-Net model of U-shape encoder-decoder architecture is widely applied in biomedical image segmentation, image denoising^39–41^, and image synthesis^42–44^. The U-Net based approach could efficiently synthesize artifact-suppressed CT-like CBCT images from CBCT images containing global scattering and local artifacts^43,44^.

To date, these deep learning-based studies have mainly focused on the improvement in voxel values of the soft tissues in CBCT images. As far as we know, no previous studies have quantitatively measured BMD from CBCT images through the improvement of the bone image using deep learning. We hypothesized that a deep learning-based method could generate QCT-like CBCT images from CBCT images for directly measuring BMD by learning the pixel-wise mapping between QCT and CBCT images. The purpose of this study was to directly and quantitatively measure BMD from CBCT images by enhancing the linearity and uniformity of the bone intensities based on a hybrid deep-learning model (QCBCT-NET) of combining the generative adversarial network (Cycle-GAN) and U-Net, and to compare the bone images enhanced by the QCBCT-NET with those by Cycle-GAN and U-Net.

## Materials and methods

### Data acquisition and preparation

**Figure 1:**
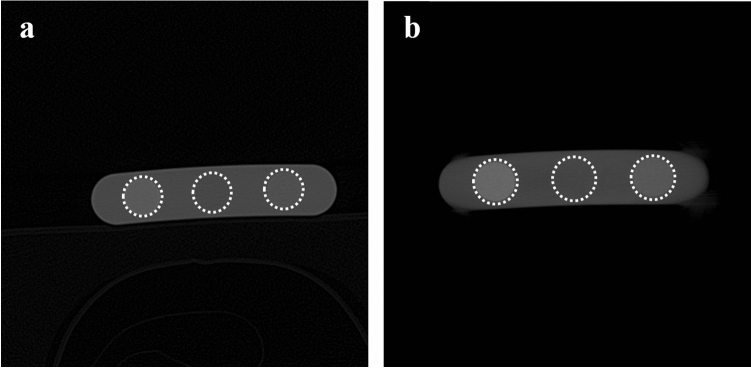
(**a**) MDCT, and (**b**) CBCT images of BMD calibration phantom with calcium hydroxyapatite inserts of three densities (0 (center circle), 100 (right circle), and 200 (left circle) mg/cm^3^).

We used two phantoms of human skulls encased in acrylic articulated for medical use (Erler Zimmer Co., Lauf, Germany), one with and the other without metal restorations causing streak artifacts. The phantoms have been used in our previous studies^45–48^. The images of the phantoms were obtained with a MDCT (Somatom Sensation 10, Siemens AG, Erlangen, Germany) and a CBCT (CS 9300, Carestream Health, Inc., Rochester, US), respectively. We acquired the CT images with voxel sizes of 0.469 × 0.469 × 0.5 mm^3^, dimensions of 512 × 512 pixels, and 16 bit depth under condition of 120 kVp and 130 mA, while the CBCT images were obtained with voxel sizes of 0.3 × 0.3 × 0.3 mm^3^, dimensions of 559 × 559 pixels, and 16 bit depth under conditions combined from 80 or 90 kVp and 8 or 10 mA. In addition, CT and CBCT images of a BMD calibration phantom (QRM-BDC Phantom 200 mm length, QRM GmbH, Moehrendorf, Germany) with calcium hydroxyapatite inserts of three densities (0 (water), 100, and 200 mg/cm^3^) were also obtained under the same condition (Fig. 1). The CT images of the skull phantoms were then converted into quantitative CT (QCT) images based on Hounsfield Units (HU) by linear calibration using the CT images of the BMD calibration phantom. The CBCT images of the skull phantoms were also converted into calibrated CBCT (CAL_CBCT) images using the corresponding images of the BMD calibration phantom for comparisons with deep learning results afterwards.

The CT image for the skull phantom was matched to the CBCT image by paired-point registration using a software (3D Slicer, MIT, Massachusetts, US), where the six landmarks were localized manually at the vertex on the lateral incisors, the buccal cusps of the first premolars, and the distobuccal cusps of the first molars^49^. The matched CT and CBCT images consisting of a matrix of 559 × 559 × 264 pixels were cropped to images of 559 × 559 × 200 pixels centered at the maxillomandibular region, and then resized to images of 256 × 256 × 200 pixels. To avoid adverse impacts from non-anatomical regions during training, binary masks were applied to the CT and CBCT images to separate the maxillomandibular region from the non-anatomical regions^44^. The binary mask images were generated by using thresholding and morphological operations. The edges of anatomical regions were extracted by applying a local range filter to the paired CBCT and CT images^50^, and the morphological operations of opening and flood fill were applied to the binarized edges obtained by thresholding to remove small blobs and fill the inner area. The corresponding CBCT and CT images were multiplied by the intersection of the two binary masks from CBCT and CT images. The voxel values outside the masked region were replaced with Hounsfield Units (HUs) of − 1000.

For deep learning, we prepared the 800 pairs of axial slice images for QCT and CBCTs from the skull phantom without metal restorations for the training and validation datasets (obtained under four conditions combined from 80 or 90 kVp, and 8 or 10 mA), and independently, another 400 pairs for QCT and CBCTs from the skull phantom with metal restorations for the test dataset (obtained under two conditions of 80 kVp and 8 mA, and 90 kVp and 10 mA).

### Hybrid deep-learning model (QCBCT-NET) for quantitative CBCT images

**Figure 2:**
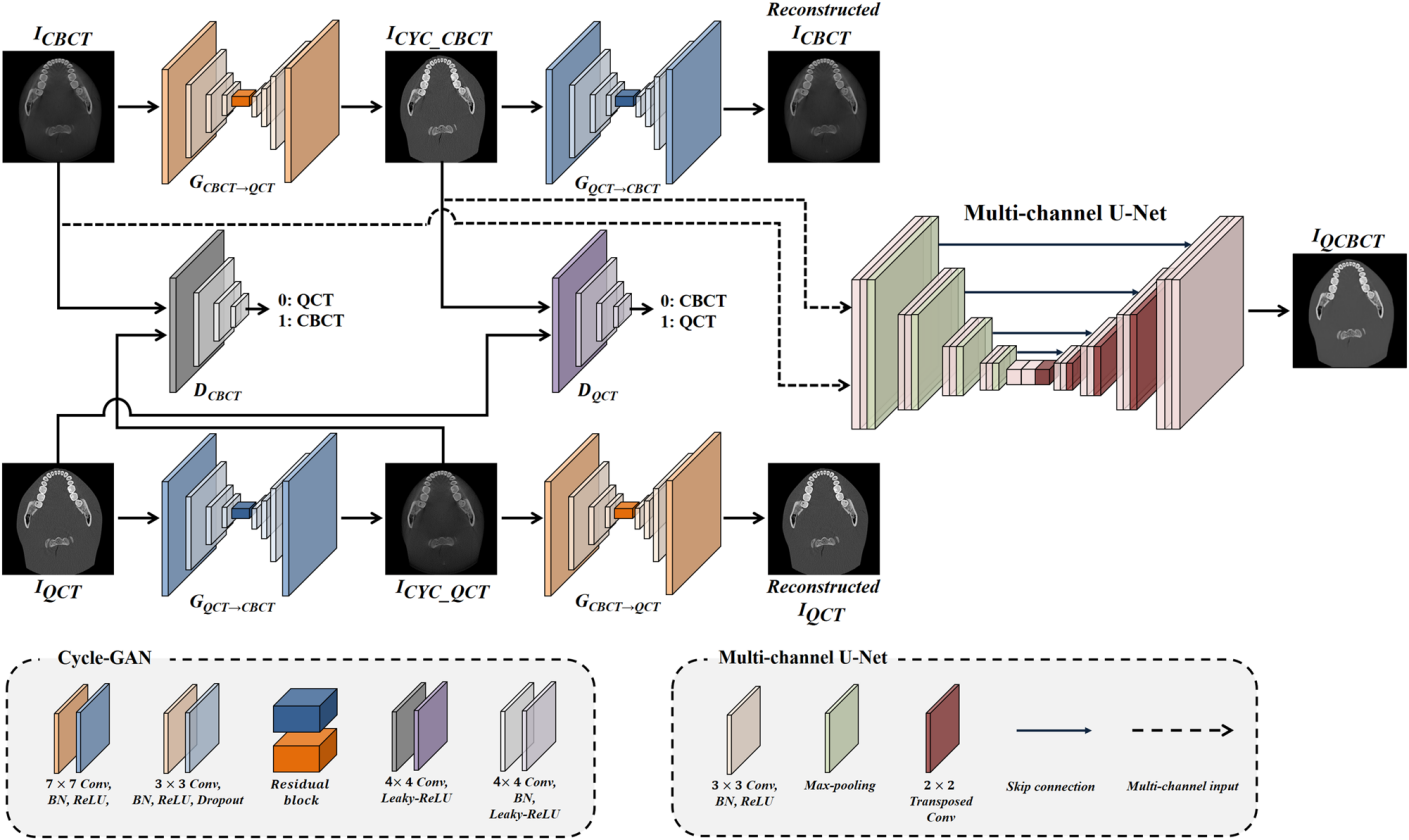
The QCBCT-NET architecture combining Cycle-GAN and the multi-channel U-net. The Cycle-GAN consisted of two generators of ${\text{G}}_{\text{CBCT}}\to {\text{QCT}}$, and ${\text{G}}_{\text{QCT}} \to {\text{CBCT}}$, and two discriminators of ${\text{D}}_{\text{CBCT}}$, and ${\text{D}}_{\text{QCT}}$. In the generators, the convolution block consisted of 7 × 7 and 3 × 3 convolution layers with batch normalization and ReLU activation, and residual blocks were embedded in the middle of the down-sampling and up-sampling layers. In discriminators, the convolution block consisted of 4 × 4 convolution layers with batch normalization and leaky ReLU activation followed by down-sampling layers. The multi-channel U-Net had two-channel inputs of CBCT and corresponding CYC_CBCT images, consisting of 3 × 3 convolution layers with batch normalization and ReLU activation, and had skip connections at each layer level. Max-pooling was used for down-sampling and transposed convolution was used for up-sampling. Consequently, the QCBCT-NET generated QCBCT images from CBCT images to quantitatively measure BMD in CBCTs.

We designed a hybrid deep-learning architecture (QCBCT-NET) consisting of Cycle-GAN and U-Net to generate QCT-like images from the conventional CBCT images (Fig. 2), and also the Cycle-GAN and the U-Net with the same architecture with QCBCT-NET, respectively, for performance comparisons. We implemented Cycle-GAN with the residual blocks^38^ combined with a multi-channel U-Net model using paired training data. The CycleGAN architecture contained two generators for yielding the CBCT to QCT (${G}_{CBCT\to QCT}$) and QCT to CBCT (${G}_{QCT\to CBCT}$) mappings, and two discriminators for distinguishing between real (${D}_{QCT}$) and generated (${D}_{CBCT}$) images. We adopted a ResNet architecture with nine residual blocks for the generators, and a PatchGAN of 70 × 70 patch for the discriminators.

The Cycle-GAN model was optimized using two part loss functions consisting of an adversarial loss and a cycle consistency loss^36^. The adversarial loss function relied on the output of the discriminators, which were defined as:

$${L}_{ADV}\left({G}_{CBCT\to QCT}\right)={{D}_{QCT}\left({I}_{QCT}\right)}^{2}+{\left({D}_{QCT}\left({G}_{CBCT\to QCT}\left({I}_{CBCT}\right)\right)-1\right)}^{2},$$

$${L}_{ADV}\left({G}_{QCT\to CBCT}\right)= {{D}_{CBCT}\left({I}_{CBCT}\right)}^{2}+{\left({D}_{CBCT}\left({G}_{QCT\to CBCT}\left({I}_{QCT}\right)\right)-1\right)}^{2},$$

where ${I}_{CBCT}$ was the CBCT image, and ${I}_{QCT}$, the QCT image.

To avoid mode collapse issues, we added a cycle consistency loss that reduced the space of mapping functions. The cycle consistency loss was defined as:

$${L}_{CYC}=\left|{G}_{QCT\to CBCT}\left({G}_{CBCT\to QCT}\left({I}_{CBCT}\right)\right)-{I}_{CBCT}\right|+\left|{G}_{CBCT\to QCT}\left({G}_{QCT\to CBCT}\left({I}_{QCT}\right)\right)-{I}_{QCT}\right|,$$

where ${I}_{CBCT}$ was the CBCT image, and ${I}_{QCT}$, the QCT image.

Finally, the loss function of Cycle-GAN was defined as:

$${L}_{GAN}= {L}_{ADV}\left({G}_{CBCT\to QCT}\right)+{L}_{ADV}\left({G}_{QCT\to CBCT}\right)+\lambda {L}_{CYC},$$

where λ controlled the relative importance of the adversarial losses, and the used value of λ was 10.

To generate QCBCT images, we implemented the multi-channel U-Net with four skip-connections between an encoder and a decoder at each resolution level using the two-channel inputs consisting of the original CBCT image, and the corresponding output of the Cycle-GAN. The multi-channel U-Net was optimized by the loss function consisting of the mean absolute difference (MAD) and structural difference (SSIM) between QCBCT and QCT images^43^, which were defined as:

$${L}_{MAD}=\left|{I}_{QCT}-{I}_{QCBCT}\right|, {L}_{\text{SSIM}}= \frac{\left({2\mu }_{QCT}{\mu }_{QCBCT}+{C}_{1}\right)\left({2\sigma }_{QCT QCBCT}+{C}_{2}\right)}{\left({{\mu }^{2}}_{QCT}+{{\mu }^{2}}_{QCBCT}+{C}_{1}\right)\left({{\sigma }^{2}}_{QCT}+{{\sigma }^{2}}_{QCBCT}+{C}_{2}\right)},$$

where ${I}_{QCBCT}$ was the QCBCT image, ${I}_{QCT}$, the QCT image, *µ*, mean, $\sigma$^2^, variance, and *C*_1_ and *C*_2_, variables to stabilize the division with weak denominators.

Finally, the loss function of the multi-channel U-Net was defined as:

$${L}_{UNet}=\left(1-\alpha \right){L}_{\text{MAD}}+\alpha \left(1-{L}_{\text{SSIM}}\right),$$

where the used value of $\alpha$ was 0.6.

The deep learning model was trained and tested using a workstation with four GPUs of Nvidia GeForce GTX 1080 Ti and 11 GB of VRAM. The Cycle-GAN model was trained by the Adam optimizer with a mini-batch size of 8 and epoch number of 200. For the first 100 epochs, the learning rate was maintained at 0.0002, and decreased linearly approaching zero for the next 100 epochs. The U-Net model was trained by the Adam optimizer with a mini-batch size of 8 and epoch number of 200. The learning rate was set to 0.0001 with momentum terms of 0.9 to stabilize the training.

To compare the performance of measuring BMD from QCBCT images produced by the QCBCT-NET with those by the Cycle-GAN or the U-Net, we used the same settings with QCBCT-NET for the Cycle-GAN and the U-Net, and trained the networks with only CBCT as the network input, respectively.

### Evaluation of quantitative CBCT images for measuring BMD

To quantitatively evaluate the performance of measuring BMD from CBCT images by the different deep learning models, we compared the mean absolute difference (MAD), peak signal to noise ratio (PSNR), normalized cross correlation (NCC), and structural similarity (SSIM) between the original QCT image (the ground truth), and QCBCT image produced by QCBCT-NET, CYC_CBCT image produced by Cycle-GAN, U_CBCT image produced by U-NET, and CAL_CBCT image produced by only calibration for the CBCT image of the test dataset obtained under two scanning conditions. The MAD was defined as the mean of the absolute differences between the intensities of the QCT and CBCT images, the PSNR as the logarithm of the maximum possible intensity (MAX) over the root mean squared error (MSE) between the intensities of the QCT and CBCT images ($PSNR=20 \times {\mathit{log}}_{10}\frac{MAX}{\sqrt{MSE}}$), the NCC as the multiplication between the intensities of the QCT and CBCT images divided by each standard deviation ($\text{NCC}=\frac{({I}_{QCT}-{\mu }_{QCT})({I}_{CBCT}-{\mu }_{CBCT})}{{\sigma }_{QCT}{\sigma }_{CBCT}}$), and SSIM the same as described above. The quantitative measurements in each slice were averaged over the whole maxilla and mandible. The higher values of PSNR, SSIM, and NCC, and the lower MAE indicated better performance for BMD measurement from CBCT images.

Spatial nonuniformity (SNU) of the CBCT images was measured as the absolute difference between the maximum and the minimum of the BMD values in rectangular ROIs around the maxilla and mandible. To evaluate the linearity of BMD measurements in the CBCT images, we analyzed the relationship between the voxel intensities of the QCT (the ground truth) and CBCT images through a linear regression of the voxel intensities (Slope, slope of linear regression) at the maxilla and mandible, respectively. The lower SNU, and the higher Slope indicated better performance for BMD measurement from CBCT images. We also performed the Bland–Altman analysis to analyze the bias and agreement limits of the BMD between QCT (the ground truth) and CBCT images at the maxilla and mandible.

We compared the performances between QCBCT and other CBCT images at the maxilla and mandible under two conditions of 80 kVp and 8 mA, and 90 kVp and 10 mA with respect to the variations of BMD values of a bone depending on their relative positions^51^, and those affected by scanning conditions. Paired two-tailed t-tests were used (SPSS v26, SPSS Inc., Chicago, IL, USA) to compare the quantitative performances between QCBCT and CYC_CBCT images, between QCBCT and U_CBCT images, and between QCBCT and CAL_CBCT images. Statistical significance level was set at 0.01.

## Results

Table 1 summarizes the means of the quantitative performance results for measuring BMD from QCBCT images produced by QCBCT-NET, CYC_CBCT produced by Cycle-GAN, U_CBCT produced by U-NET, and CAL_CBCT produced by calibration for the CBCT images of test datasets acquired for the skull phantom with metal restorations under conditions of 80 kVp and 8 mA, and 90 kVp and 10 mA. The BMD images of QCBCTs significantly outperformed the CYC_CBCT and U_CBCT images in MAD, PSNR, SSIM, and NCC at both the maxilla and mandible area when compared to the original QCT images (Table 1). All performances from the QCBCT images exhibited significant differences with those from the CYC_CBCT or U_CBCT images at the maxilla and mandible (p < 0.01) except for the SNU from the U_CBCT (p = 0.04) (Table 1). Compared to the BMD measurements from the CYC_CBCT image, the BMD from the QCBCT showed increases of 38% MAD, 20% PSNR, 45% SSIM, 40% NCC, 80% SNU, and 84% Slope at the maxilla, and 39% MAD, 20% PSNR, 50% SSIM, 40% NCC, 47% SNU, and 102% Slope at the mandible for CBCT images under condition of 80 kVp and 8 mA (Table 2). Compared to the BMD measurement from the U_CBCT image, increases of 59% MAD, 41% PSNR, 112% SSIM, 58% NCC, -17% SNU, and 167% Slope at the maxilla, and 49% MAD, 33% PSNR, 81% SSIM, 54% NCC, -25% SNU, and 142% Slope at the mandible for CBCT images under condition of 80 kVp and 8 mA (Table 2). Under the higher dose condition of 90 kVp and 10 mA, the BMD from the QCBCT also showed higher performances at both the maxilla and mandible compared to the CYC_CBCT and U_CBCT (Table 2). Therefore, the BMDs from the QCBCT demonstrated more accuracy than those from the CYC_CBCT and U_CBCT without regard to relative positions of the bone, or effects from different scanning conditions.

**Table 1:** Quantitative performance of CBCT images produced by QCBCT-NET, Cycle-GAN, U-Net, and CAL_CBCT compared to the original QCT images for measuring BMD values at the maxilla (1–81 slices) and mandible (82–200 slices) for test datasets under conditions of 80 kVp and 8 mA, and 90 kVp and 10 mA.

		Maxilla						Mandible					
		MAD	PSNR	SSIM	NCC	SNU	Slope	MAD	PSNR	SSIM	NCC	SNU	Slope
80 kVp 8 mA	QCBCT-NET	203.45 ± 27.24*^†‡^	23.87 ± 1.34*^†‡^	0.87 ± 0.02*^†‡^	0.87 ± 0.02*^†‡^	15.60 ± 7.85^†‡^	0.83 ± 0.04*^†‡^	190.79 ± 34.46*^†‡^	24.58 ± 1.39*^†‡^	0.87 ± 0.07*^†‡^	0.88 ± 0.06*^†‡^	21.85 ± 7.72^†‡^	0.85 ± 0.16*^†‡^
	Cycle-GAN (p-value)	328.91 ± 55.12 (0.00)	19.94 ± 1.63 (0.00)	0.60 ± 0.07 (0.00)	0.62 ± 0.08 (0.00)	79.04 ± 13.48 (0.00)	0.45 ± 0.06 (0.00)	313.14 ± 58.68 (0.00)	20.52 ± 1.42 (0.00)	0.58 ± 0.08 (0.00)	0.63 ± 0.11 (0.00)	41.59 ± 10.56 (0.00)	0.42 ± 0.09 (0.00)
	U-Net (p-value)	493.91 ± 45.14 (0.00)	16.93 ± 0.86 (0.00)	0.41 ± 0.07 (0.00)	0.55 ± 0.08 (0.00)	13.39 ± 3.22 (0.04)	0.31 ± 0.06 (0.00)	371.00 ± 36.81 (0.00)	18.54 ± 1.31 (0.00)	0.48 ± 0.08 (0.00)	0.57 ± 0.12 (0.00)	17.54 ± 2.84* (0.00)	0.35 ± 0.08 (0.00)
	CAL_CBCT (p-value)	592.40 ± 53.76 (0.00)	15.63 ± 0.80 (0.00)	0.31 ± 0.08 (0.00)	0.61 ± 0.08 (0.00)	69.30 ± 15.05 (0.00)	0.26 ± 0.06 (0.00)	491.44 ± 95.51 (0.00)	17.33 ± 1.52 (0.00)	0.40 ± 0.05 (0.00)	0.62 ± 0.11 (0.00)	39.19 ± 11.14 (0.00)	0.30 ± 0.08 (0.00)
90 kVp 10 mA	QCBCT-NET	265.4 ± 63.41*^†‡^	21.92 ± 1.98*^†‡^	0.79 ± 0.02*^†‡^	0.84 ± 0.02*^†‡^	27.09 ± 38.42^†‡^	0.62 ± 0.04*^†‡^	236.25 ± 68.62*^†‡^	22.98 ± 2.36*^†‡^	0.79 ± 0.08*^†‡^	0.80 ± 0.15*^†‡^	15.87 ± 4.24^†‡^	0.66 ± 0.11*^†‡^
	Cycle-GAN (p-value)	296.82 ± 53.03 (0.00)	21.08 ± 1.39 (0.00)	0.72 ± 0.04 (0.00)	0.76 ± 0.05 (0.00)	68.91 ± 47.76 (0.00)	0.55 ± 0.05 (0.00)	288.28 ± 61.30 (0.00)	21.38 ± 2.17 (0.00)	0.69 ± 0.07 (0.00)	0.71 ± 0.14 (0.00)	36.22 ± 8.96 (0.00)	0.53 ± 0.09 (0.00)
	U-Net (p-value)	474.15 ± 52.87 (0.00)	17.40 ± 0.88 (0.00)	0.50 ± 0.06 (0.00)	0.68 ± 0.09 (0.00)	16.02 ± 30.39* (0.00)	0.38 ± 0.04 (0.00)	370.59 ± 104.16 (0.00)	19.66 ± 2.68 (0.00)	0.57 ± 0.07 (0.00)	0.67 ± 0.14 (0.00)	12.80 ± 4.08* (0.00)	0.40 ± 0.06 (0.00)
	CAL_CBCT (p-value)	661.48 ± 61.59 (0.00)	14.87 ± 0.78 (0.00)	0.29 ± 0.08 (0.00)	0.75 ± 0.05 (0.00)	52.71 ± 23.00 (0.00)	0.31 ± 0.05 (0.00)	573.25 ± 93.37 (0.00)	16.15 ± 1.42 (0.00)	0.37 ± 0.08 (0.00)	0.72 ± 0.13 (0.00)	72.44 ± 30.46 (0.00)	0.31 ± 0.06 (0.00)

*MAD* mean absolute difference, *PSNR* peak signal to noise ratio, *SSIM* structural similarity, *NCC* normalized cross correlation, *SNU* spatial nonuniformity, *Slope* slope of linear regression between the voxel intensities.

Mean ± SD.

*Significant difference (*p* < 0.01) between QCBCT-NET and U-Net, ^†^(*p* < 0.01) between QCBCT-NET and Cycle-GAN, and ^‡^(*p* < 0.01) between QCBCT-NET and CAL_CBCT.

**Table 2:** Percentage increases of QCBCT-NET performance compared to Cycle-GAN and U-Net for measuring BMD values at the maxilla (1–81 slices) and mandible (82–200 slices) for CBCT images of test datasets under conditions of 80 kVp and 8 mA, and 90 kVp and 10 mA.

		Maxilla (%)						Mandible (%)					
		MAD	PSNR	SSIM	NCC	SNU	Slope	MAD	PSNR	SSIM	NCC	SNU	Slope
80 kVp 8 mA	vs. Cycle-GAN	38.14	19.71	45.00	40.32	80.26	84.44	39.07	19.79	50.00	39.68	47.46	102.38
	vs. U-Net	58.81	40.99	112.20	58.18	− 16.50	167.74	48.57	32.58	81.25	54.39	− 24.57	142.86
90 kVp 10 mA	vs. CycleGAN	10.59	3.98	9.72	10.53	59.10	12.73	17.69	7.48	14.49	12.68	56.18	24.53
	vs. U-Net	44.03	25.98	58.00	23.53	− 73.47	63.16	36.40	16.89	38.60	19.40	− 23.98	65.00

*MAD* mean absolute difference, *PSNR* peak signal to noise ratio, *SSIM* structural similarity, *NCC* normalized cross correlation, *SNU* spatial nonuniformity, *Slope* slope of linear regression between the voxel intensities.

**Figure 3:**
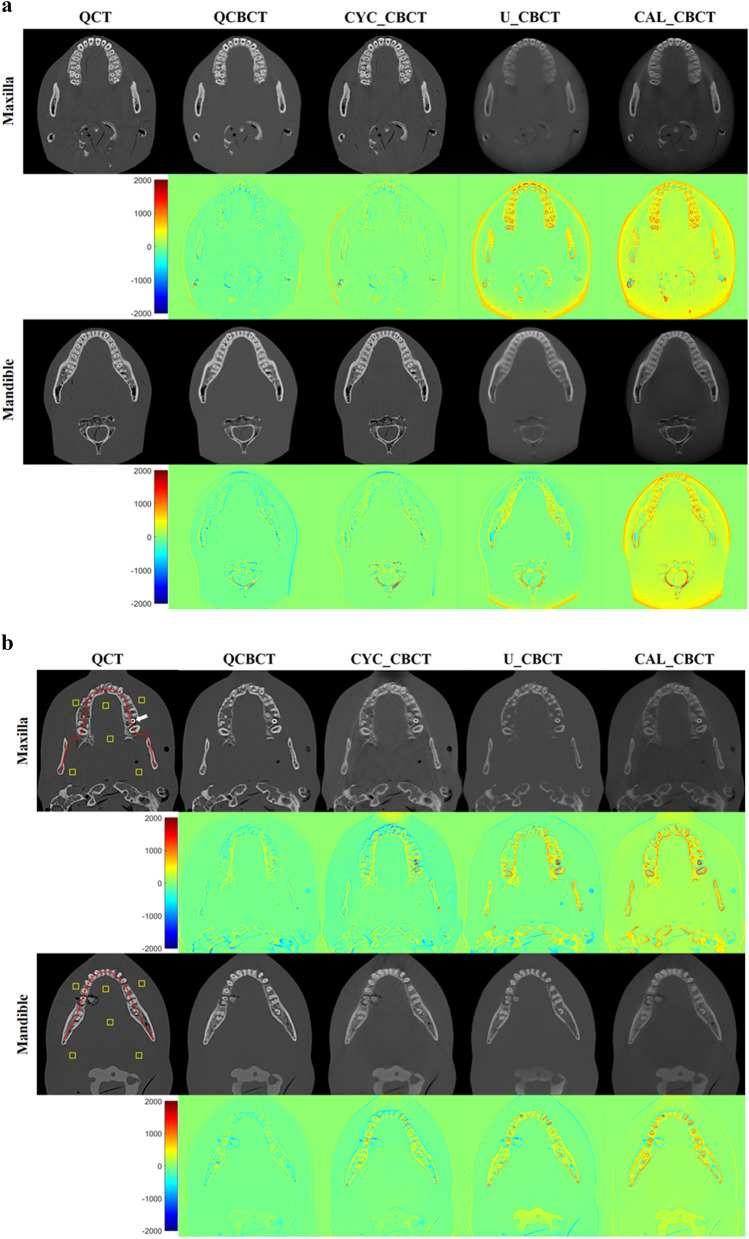

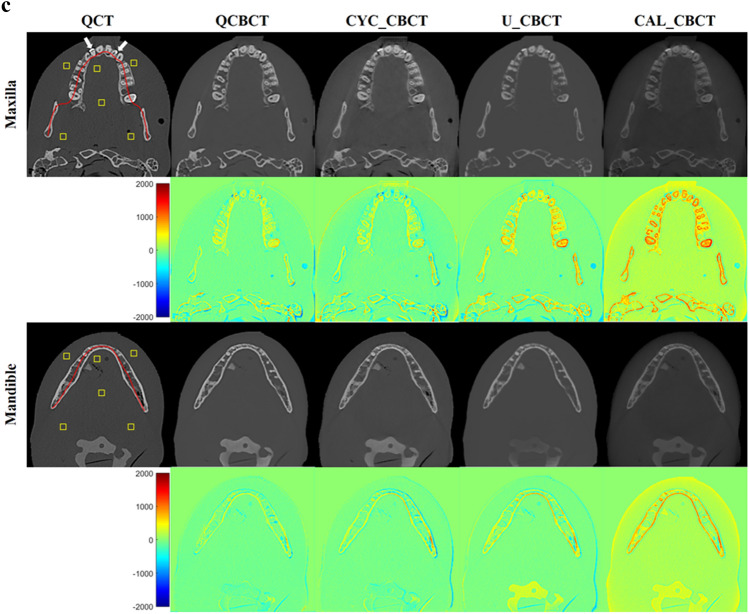
The axial slices of BMD images from the original QCT, their generations by deep learning methods (the first and third row), and their subtractions from the original QCT images (the second and fourth row) at the maxilla and the mandible. QCBCT produced by QCBCT-NET, CYC_CBCT by Cycle-GAN, U_CBCT by U-NET, and CAL_CBCT by only calibration from (**a**) training datasets under condition of 90 kVp and 10 mA, (**b**) test datasets under condition of 80 kVp and 8 mA, and (**c**) test datasets under condition of 90 kVp and 10 mA. The yellow squares shown in the QCT image were ROIs for calculation of the spatial nonuniformity (SNU), the red curve shown in the QCT image was the dental arch for BMD (voxel intensity) profiles, and the white arrows shown in the QCT images indicated the dental implant at the maxilla in (**b**), and the dental restorations at the maxilla in (**c**).

Figure 3 shows the axial slices of the BMD images from the original QCT, QCBCT, CYC_CBCT, U_CBCT, and CAL_CBCT at the maxilla and mandible. As shown in the subtraction images in Fig. 3, the BMD image quality of the QCBCTs for the two regions exhibited substantial improvement over those of CYC_CBCT, U_CBCT, and CAL_CBCT in terms of BMD (voxel intensity) differences compared to the original QCT images. The large differences around the teeth and dense bone of higher voxel intensities (BMD) seen in the CAL_CBCT were more reduced in the QCBCT than in the CYC_CBCT or U_CBCT images.

**Figure 4:**
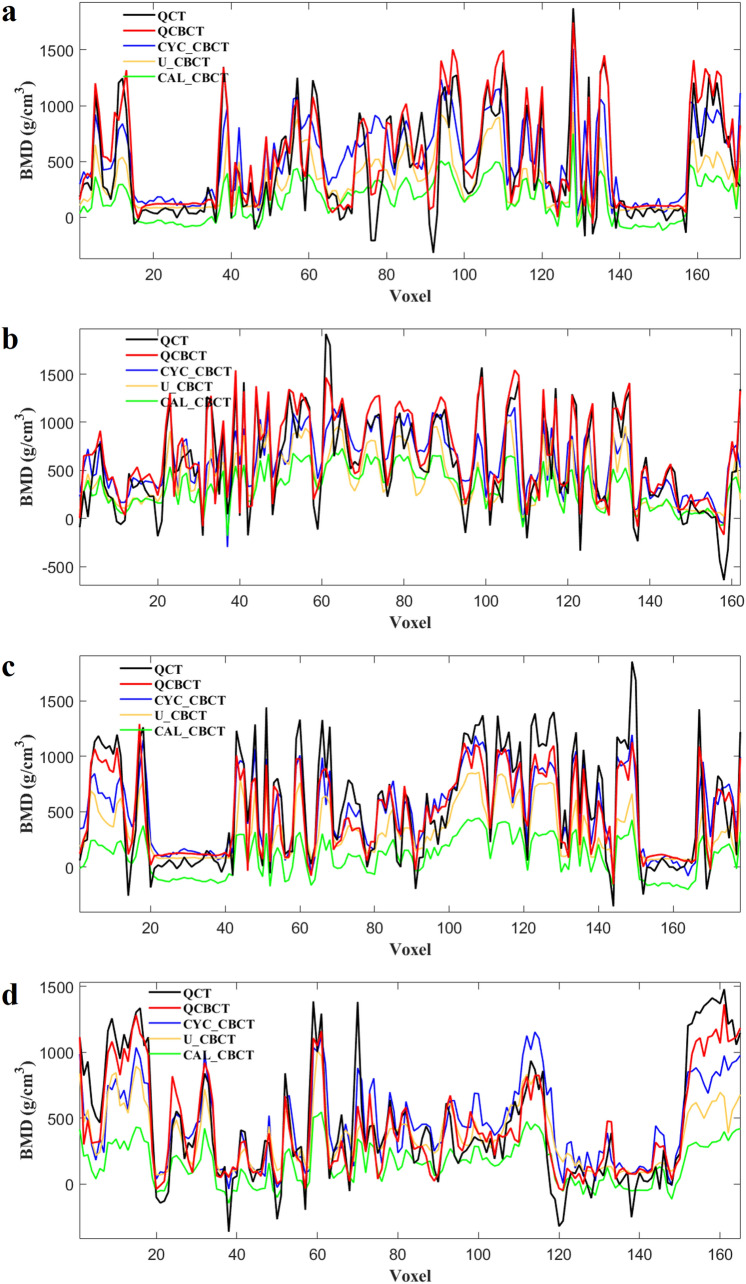
The BMD (voxel intensity) profiles along the dental arch at the maxilla and the mandible in the QCT, and QCBCT, CYC_CBCT, U_CBCT, and CAL_CBCT images shown in Fig. 2. Pearson correlation coefficients of QCBCT, CYC_CBCT, U_CBCT, and CAL_CBCT with the original QCT were (**a**) 0.92, 0.65, 0.60, and 0.65, respectively, for the profile at the maxilla and, (**b**) 0.93, 0.70, 0.65, and 0.69, respectively, for the profile at the mandible shown in Fig. 2b, and (**c**) 0.92, 0.89, 0.84, and 0.88, respectively, for the profile at the maxilla, and (**d**) 0.93, 0.81, 0.82, and 0.82, respectively, for the profile at the mandible shown in Fig. 2c.

**Figure 5:**
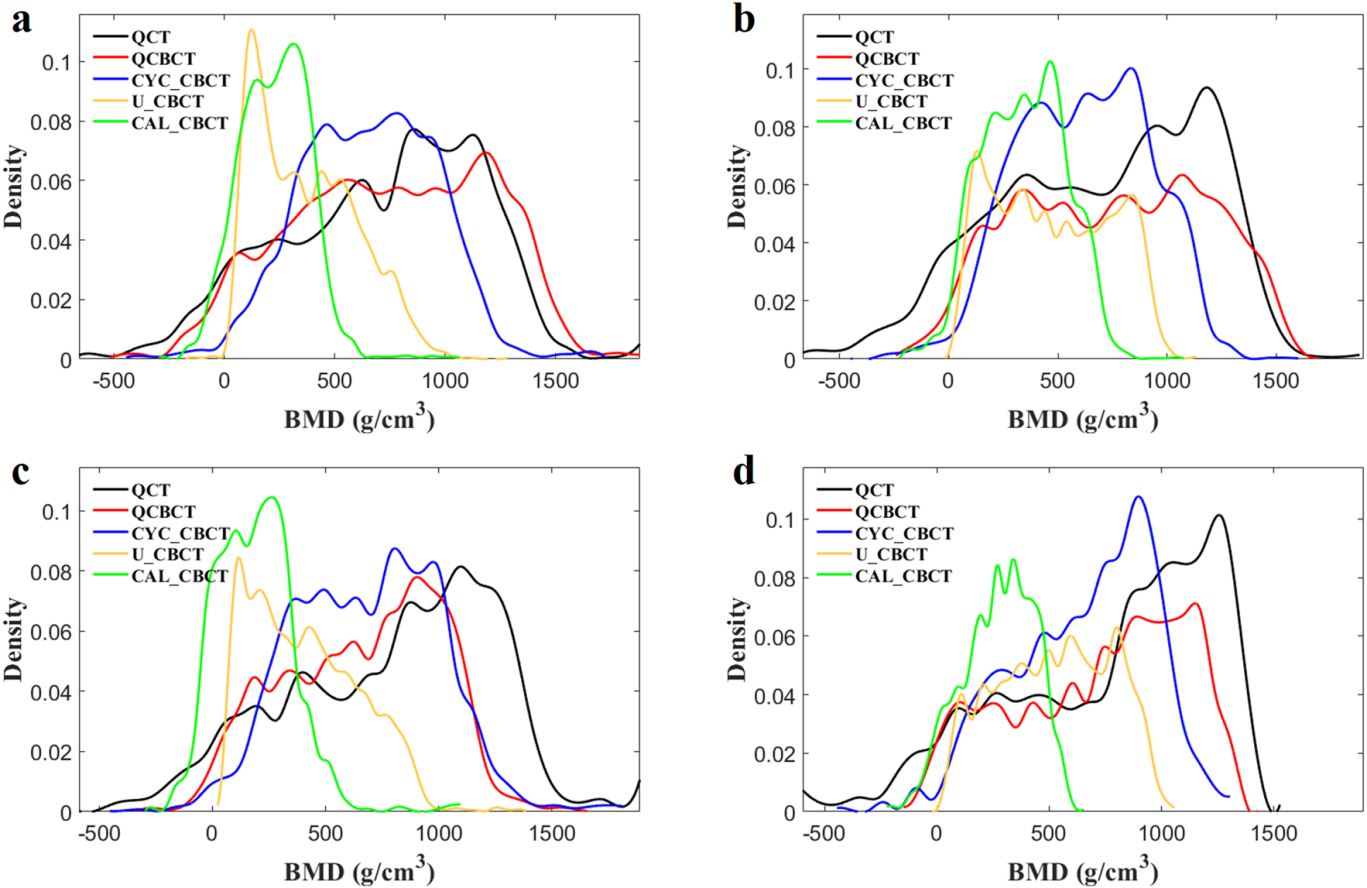
The BMD distribution in an axial slice of the original QCT, and QCBCT, CYC_CBCT, U_CBCT, and CAL_CBCT images. (**a**) CBCT images at the maxilla under condition of 80 kVp and 8 mA, (**b**) at the mandible under condition of 80 kVp and 8 mA, (**c**) at the maxilla under condition of 90 kVp and 10 mA, and (**d**) at the mandible under condition of 90 kVp and 10 mA.

**Figure 6:**
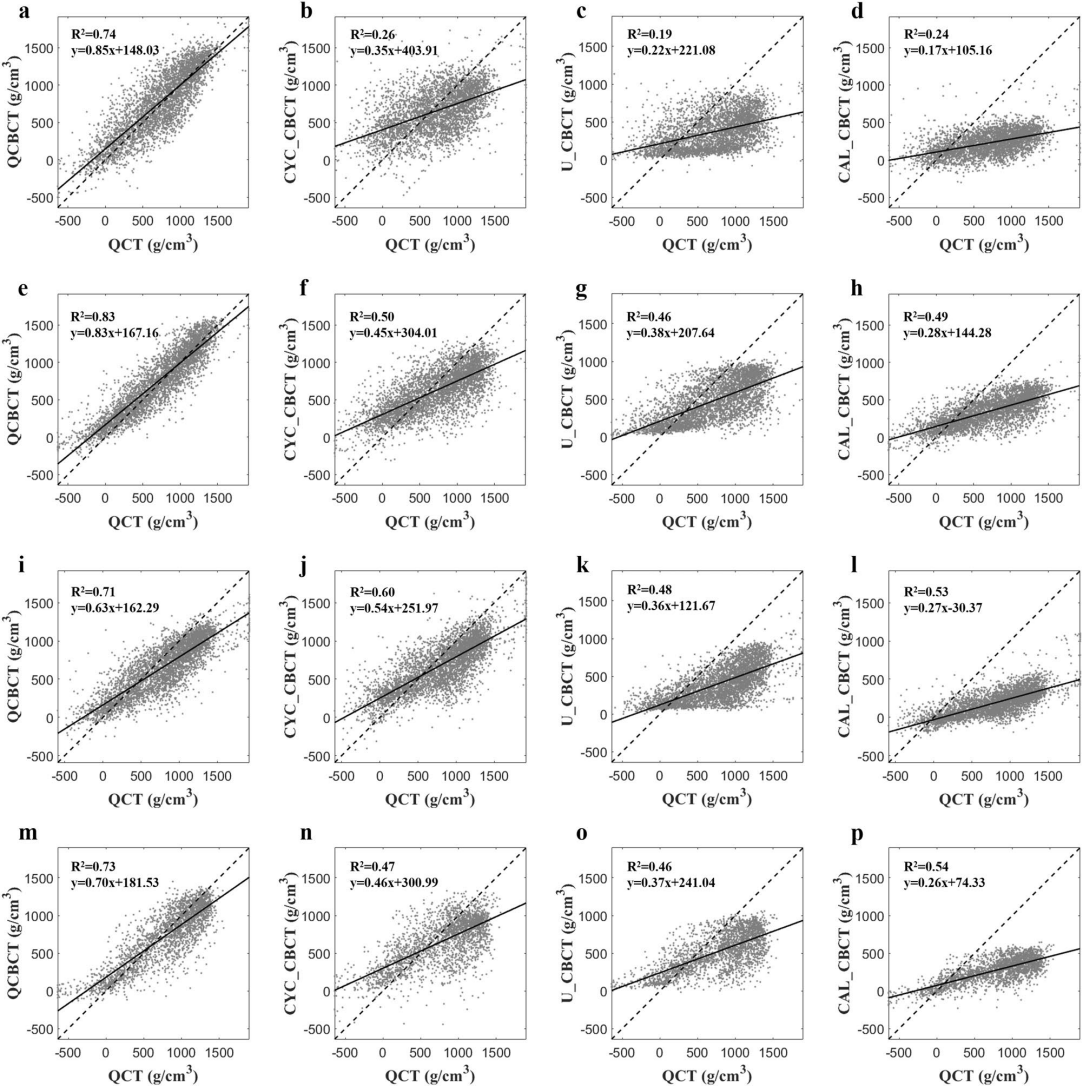
The linear relationships between the original QCT, and QCBCT, CYC_CBCT, U_CBCT, and CAL_CBCT images. (**a–d**) CBCT images at the maxilla under condition of 80 kVp and 8 mA, (**e–h**) at the mandible under condition of 80 kVp and 8 mA, (**i–l**) at the maxilla under condition of 90 kVp and 10 mA, and (**m–p**) at the mandible under condition of 90 kVp and 10 mA.

**Figure 7:**
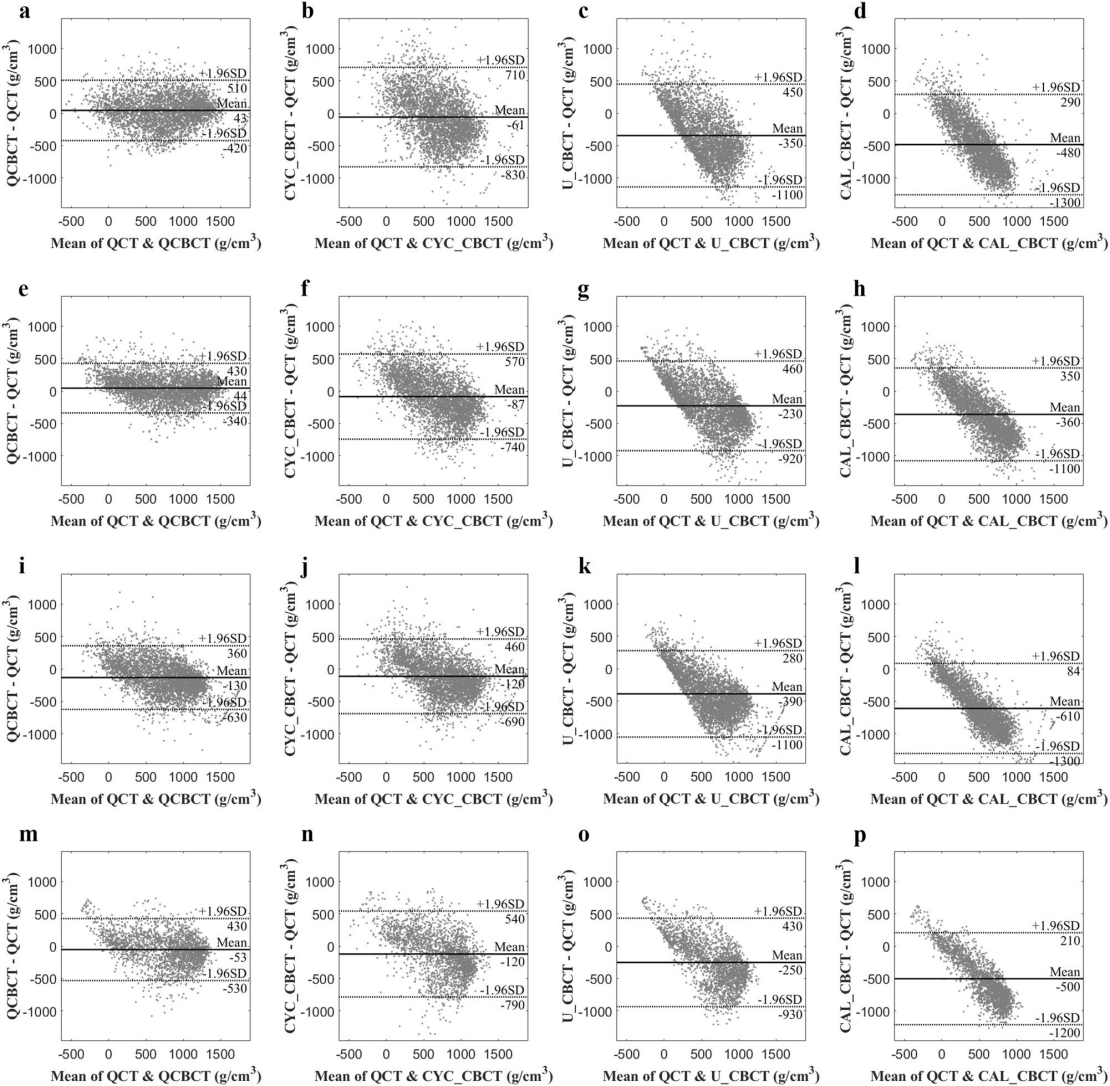
The Bland–Altman plots between the original QCT, and QCBCT, CYC_CBCT, U_CBCT, and CAL_CBCT images. (**a–d**) CBCT images at the maxilla under condition of 80 kVp and 8 mA, (**e–h**) at the mandible under condition of 80 kVp and 8 mA, (**i–l**) at the maxilla under condition of 90 kVp and 10 mA, and (**m–p**) at the mandible under condition of 90 kVp and 10 mA.

Figure 4 shows the BMD (voxel intensity) profiles that were acquired along the dental arch at the maxilla and mandible in the QCT and CBCT images as shown in Fig. 3. The BMD profile from the QCBCT images more closely reflected the original QCT than the CYC_CBCT and U_CBCT images with higher correlations with the QCT than other CBCT images, although the dental implant and restorations showed higher voxel intensities compared to other anatomical structures (Fig. 4). Therefore, the QCBCT image exhibited more improved structural preservation and edge sharpness of the bone than the CYC_CBCT and U_CBCT images at both the maxilla and mandible. The BMD distribution of the QCBCT also more closely restored the original QCT than that of the CYC_CBCT and U_CBCT images in an axial slice at the maxilla and mandible (Fig. 5). The linear relationship between the QCT and QCBCT images showed more contrast and correlation than that between QCT and other CBCT images with the larger slope and better goodness of fit (Fig. 6). The Bland–Altman plot between QCT and QCBCT images also showed higher linear relationships and better agreement limits than that between QCT and other CBCT images (Fig. 7). Therefore, the QCBCT images showed more improvement in preservation for the original distribution and linear relationship of the BMD values compared to CYC_CBCT and U_CBCT images.

## Discussion

We developed a hybrid deep-learning model (QCBCT-NET) consisting of Cycle-GAN and U-Net to quantitatively and directly measure BMD from CBCT images. The BMD measurements of QCBCT images produced by QCBCT-NET significantly outperformed the CYC_CBCT images produced by Cycle-GAN and U_CBCT images produced by U-Net at both the maxilla and mandible area when compared to the original QCT. We used paired training data in the Cycle-GAN implementation with the residual blocks, which forced the network to focus on reducing image artifacts and enhancing bone contrast, rather than focusing on bone structural mismatches. Through the residual blocks in the generator architecture of the Cycle-GAN, the network could learn the difference between the source and target based on the residual image and generate corrected bone images more accurately^52^. In a study, a Cycle-GAN was used to capture the relationship from CBCT to CT images while simultaneously supervising an inverse of the CT to CBCT transformation model^36^. The Cycle-GAN doubled the process of a typical GAN by enforcing an inverse transformation, which doubly constrained the model and increased accuracy in the output images^38^. In our study, the Cycle-GAN can learn both intensity and textural mapping from a source distribution of the CBCT bone image to a target distribution of the QCT bone image.

In previous studies, U-Net architectures were used to directly synthesize CT-like CBCT images for their corresponding CT images especially on paired datasets^43,44^. The U-Net could suppress global scattering artifacts and local artifacts derived from CBCT images by capturing both global and local features in the image spatial domain^43^. In addition, the spatial uniformity of CT-like CBCT images was enhanced close to those of corresponding CT images while maintaining the anatomical structures on the CBCT images^44^. Therefore, in our results, the spatial uniformity of CBCT images produced by U-Net was improved, but the contrast of the bone images was reduced when compared to the CYC_CBCT images by Cycle-GAN.

In our study, the two-channel U-Net, which learned spatial information of CBCTs and corresponding CYC_CBCT images simultaneously, could improve image contrast and uniformity by suppressing beam hardening artifacts and scattering noise^43^. The CYC_CBCT images out of the two inputs helped the U-Net to focus on learning pixel-wise correspondence (or mapping) between QCT and CBCT images while maintaining the original intensity distribution of the bone structures. The combination loss of MAE and SSIM in the U-Net facilitated faster convergence and better accuracy considering the pixel-wise errors and structural similarity. As a result, the BMDs (voxel intensities) from the QCBCT demonstrated more accuracy than those from the CYC_CBCT and U_CBCT without regard to relative positions of the bone in the image volume^51^, or effects from different radiation doses or scanning conditions used in clinical settings.

We combined the Cycle-GAN with the two-channel U-Net model to further improve the contrast and uniformity of the CBCT bone images. The Cycle-GAN improved the contrast of the bone images by reflecting the original BMD distribution of the QCT images locally, while the two-channel U-Net improved the spatial uniformity of the bone images by globally suppressing the image artifacts and noise. As a result, the Cycle-GAN and two-channel U-Net worked to provide complementary benefits in improving the contrast and uniformity of the bone image locally and globally. Consequently, the QCBCT-NET could substantially enhance the linearity, uniformity, and contrast as well as the anatomical and quantitative accuracy of the bone images in order to quantitatively measure BMD in CBCT. Although the BMD linear relationships and agreement limits of QCBCT images were superior to those of CYC_CBCT and U_CBCT images, the accuracy of our method should be further improved for clinical applications.

Our study had some limitations. First, because paired CBCT and CT images were acquired at different imaging situations typically, the bone structures of the images were not perfectly aligned even after registration. Therefore, the registration error of CBCT and CT images might cause adverse impacts during network training. Second, our study had a potential limitation of generalization ability due to using a relatively small number of training dataset. Overfitting of the training CNN model, which resulted in the model learning statistical regularity specific to the training dataset, could impact negatively the model’s ability to generalize to a new dataset^53^. Third, the results presented in this study were based on two human skull phantoms with and without metal restorations instead of actual patients. Our method needs to be validated for the dataset from actual patients having dental fillings and restorations for its application in clinical research and practice, and compared to the conventional scatter-based method in future studies.

## Conclusions

We proposed QCBCT-NET to directly and quantitatively measure BMD from CBCT images based on a hybrid deep-learning model of combining the generative adversarial network (GAN) and U-Net. The Cycle-GAN and two-channel U-Net in QCBCT-Net provided complementary benefits of improving the contrast and uniformity of the bone image locally and globally. The BMD images produced by QCBCT-NET significantly outperformed the images produced by Cycle-GAN or U-Net in MAD, PSNR, SSIM, NCC, and linearity when compared to the original QCT. The QCBCT-NET substantially enhanced the linearity, uniformity, and contrast as well as the anatomical and quantitative accuracy of the bone images, and demonstrated more accuracy than the Cycle-GAN and the U-Net for quantitatively measuring BMD in CBCT. In future studies, we plan to evaluate the proposed method on the actual patient dataset to prove its clinical efficacy.

## Data Availability

The datasets generated during and/or analyzed during the current study are available from the corresponding author on reasonable request.
